# Migration-driven microbial adaptation and ecological spillover in birds

**DOI:** 10.1093/ismeco/ycag042

**Published:** 2026-03-10

**Authors:** Amina Tufail, Tingbei Bo, Na Zhao, Jundong Duan, Jianshi Jin, Bushra Nisar Khan, Yanhua Qu, Song Gang, Lei Fumin

**Affiliations:** State Key Laboratory of Animal Biodiversity Conservation and Integrated Pest Management, Institute of Zoology, Chinese Academy of Sciences, 1 Beichen West Road, Chaoyang District, Beijing 100101, P. R. China; College of Life Sciences, University of Chinese Academy of Sciences, Beijing 101408, China; School of Grassland Science, Beijing Forestry University, Beijing 100083, China; The Anhui Provincial Key Laboratory of Biodiversity Conservation and Ecological Security in the Yangtze River Basin, College of Life Sciences, Anhui Normal University, Wuhu 24100, China; State Key Laboratory of Animal Biodiversity Conservation and Integrated Pest Management, Institute of Zoology, Chinese Academy of Sciences, 1 Beichen West Road, Chaoyang District, Beijing 100101, P. R. China; College of Life Sciences, University of Chinese Academy of Sciences, Beijing 101408, China; State Key Laboratory of Animal Biodiversity Conservation and Integrated Pest Management, Institute of Zoology, Chinese Academy of Sciences, 1 Beichen West Road, Chaoyang District, Beijing 100101, P. R. China; Institute of Zoology, University of the Punjab, Lahore 54590, Pakistan; State Key Laboratory of Animal Biodiversity Conservation and Integrated Pest Management, Institute of Zoology, Chinese Academy of Sciences, 1 Beichen West Road, Chaoyang District, Beijing 100101, P. R. China; State Key Laboratory of Animal Biodiversity Conservation and Integrated Pest Management, Institute of Zoology, Chinese Academy of Sciences, 1 Beichen West Road, Chaoyang District, Beijing 100101, P. R. China; Hainan Institute of National Park, Haikou 570203, China; State Key Laboratory of Animal Biodiversity Conservation and Integrated Pest Management, Institute of Zoology, Chinese Academy of Sciences, 1 Beichen West Road, Chaoyang District, Beijing 100101, P. R. China

**Keywords:** antimicrobial resistance (AMR), bird migration, bursa of Fabricius, gut microbiota remodeling, gut microbial symbiosis, host–microbiota co-adaptation, microbial spillover, One Health, zoonotic pathogens

## Abstract

Migratory birds perform one of the most physiologically demanding feats in the animal kingdom, rapidly accumulating fat reserves and enduring extreme environmental and immunological stress. Central to their survival is the gut microbiota, a diverse assemblage of microorganisms that contributes to energy harvesting, immune modulation, and host adaptation. As birds traverse varied landscapes and feed on diverse diets, their gut microbial communities undergo marked compositional and functional shifts. These changes can optimize nutrient absorption and immune preparedness, but they may also lead to dysbiosis under conditions of stress or pathogen exposure, potentially impairing migratory performance. Importantly, migratory birds also act as mobile reservoirs of zoonotic pathogens and antimicrobial resistance genes. Stopover sites, critical refueling points along migratory routes, serve as hubs for microbial exchange between wild birds, domestic animals, and human-altered environments, thereby amplifying spillover risks. We highlight current gaps in understanding the forces that remodel the gut microbiota and mechanistic links between microbiota dynamics and migratory performance, and propose integrative research strategies involving longitudinal sampling, meta-omics, and controlled experiments. Ultimately, bird migration offers a powerful model for exploring host–microbe co-adaptation under extreme ecological pressures. Addressing these dynamics through a One Health framework is essential for biodiversity conservation, disease mitigation, and global health security.

## Introduction

The gut microbiota, often called the host’s second genome, performs vital functions that significantly impact the host [1, 2]. Interactions between the host and its gut microbiota regulate physiological processes like metabolism (digestion, nutrient absorption) [3–5], immune system function [6, 7], and protection against pathogens [8]. The host, in return, provides microbes with a stable habitat and nutrients and facilitates their environmental dispersal via fecal and feather shedding [9, 10]. Birds host diverse and intricate communities of microorganisms, which are found both internally (in the gastrointestinal and reproductive tracts) and externally (on skin and feathers) [11, 12].

Migration is an adaptive life-history strategy that enables birds to exploit spatially and temporally variable resource changes [13]. The complex phenomenon of migration is influenced by factors such as vectors for pathogens and antibiotic-resistant microbes, especially due to anthropogenic environmental changes [14]. Concerns about pathogen spread have sparked investigations into antimicrobial resistance (AMR) genes carried by migratory birds [15, 16]. AMR genes have been detected in migratory bird gut microbiota, with cloacal viromes also showing high viral diversity [17]. Stopover sites, where diverse species congregate, are hotspots for microbial spillover, especially near anthropogenic habitats [18, 19]. For example, 75% of sampled shorebirds in Delaware Bay carried *Salmonella* strains also found in poultry, and CTX-M-15 (a critical AMR gene) was detected in 30% of Arctic-breeding geese [20]. Such horizontal microbial spillover events raise serious concerns for global public health, wildlife conservation, and ecosystem integrity [18, 21]. Understanding how migration influences microbial community assembly and spillover potential is thus imperative within the One Health framework. Furthermore, analyses of migratory birds’ gut microbiota have important applications in their conservation practices [22, 23].

Currently, there is a lack of comprehensive studies linking gut microbiota adaptations and ecological spillovers to specific migratory phases. Moreover, the impact of environmental factors at stopover sites on microbiota composition remains underexplored. Addressing these gaps can provide new insights into conservation and disease mitigation. This review synthesizes current knowledge on migration as a driver of gut microbial adaptations and accelerator of ecological spillover. We propose a conceptual framework emphasizing that (i) migration imposes selective pressures favoring gut microbes optimizing host metabolism, immunity, and oxidative stress management, resulting in a dynamic, phase-dependent core microbiota. (ii) Migratory birds act as long-distance vectors of zoonotic pathogens and AMR genes, alongside other transmission routes. Stopover sites serve as key microbial exchange hubs linking wildlife, domestic animals, and humans. (iii) There is a need to identify key research gaps and to emphasize longitudinal, mechanistic, and multi-omic studies capable of separating migration-specific effects from confounding influences such as diet, phylogeny, season, and environmental exposure.

## Microbial community remodeling across the migratory cycle

Seasonal migration reshapes the avian gut microbiota through changes in diet, geography, physiology, and microbial exposure. This remodeling is not merely ecological turnover but a regulated, adaptive process. Recent studies highlight microbiota’s plasticity, with shifts occurring over short timescales [24–28]. Across multiple avian species, empirical evidence shows that α-diversity often decreases during energetically demanding flight phases and partially rebounds at stopovers, although the magnitude and direction of these changes vary depending on diet, species ecology, and environmental exposure, highlighting that migration interacts with multiple ecological drivers rather than operating as a single dominant force [26, 29, 30]. These diversity shifts may influence host energy metabolism, immunity, and pathogen defense [31]. Together, these shifts reflect how seasonal migration orchestrates microbiota changes aligned with energy demands and immune pressures, and habitat exposures. The extent of these patterns varies across taxa, emphasizing that migration-driven microbiome remodeling reflects species-specific ecology rather than a uniform rule.

### Compositional shifts across different migratory phases

**Figure 1:**
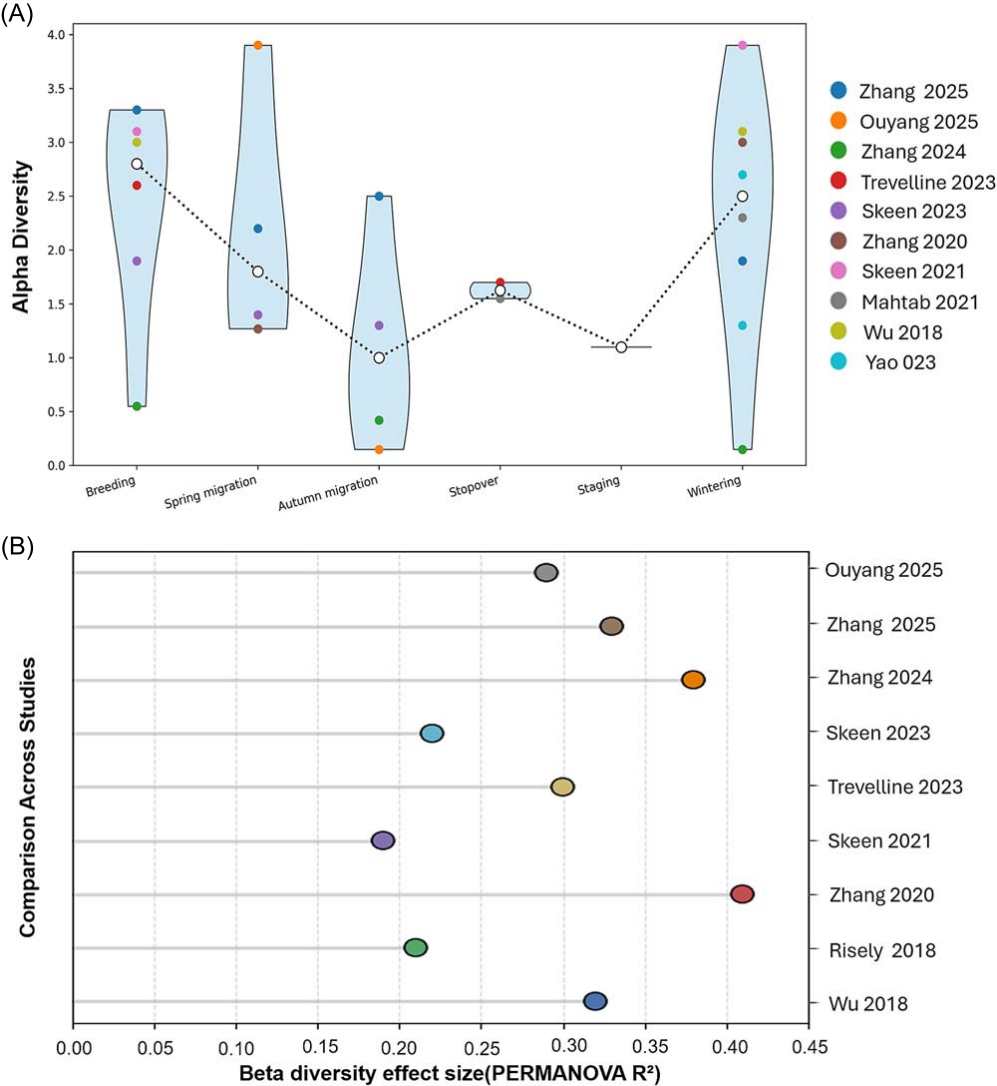
Migration-associated restructuring of avian gut microbiota across migratory phases. (A) Alpha-diversity meta-trajectory synthesized from published studies. Violin plots summarize alpha-diversity values manually extracted from the literature across breeding, spring migration, autumn migration, stopover, staging, and wintering phases. Points represent individual studies, and the dotted line indicates phase-wise median trends, revealing reduced alpha diversity during migratory flight phases and partial recovery at stopover and wintering sites. (B) Beta-diversity synthesis across migratory phases based on reported PERMANOVA results from published studies. Points represent study-level effect sizes (*R*^2^), illustrating consistent, phase-dependent shifts in microbial community composition during migration. Together, panels demonstrate systematic microbiome remodeling associated with migratory transitions.

**Figure 2:**
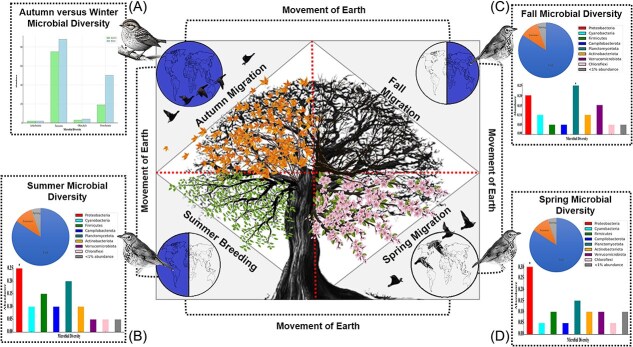
Seasonal variation in gut microbiota of migratory birds. The figure is adapted with permission: 7(a) [172], 7(b), (c), (d) [240]. This figure depicts temporal shifts in gut microbial composition during different stages of the migratory cycle: spring migration, summer breeding, autumn migration, and wintering. The central circular tree diagram highlights the cyclical nature of migration, with each quadrant representing a season. Panels (A)–(D) illustrate microbial diversity at key phases: (A) comparison of autumn and winter migration, showing a pronounced seasonal shift with higher microbial abundance in winter; (B, C) summer and spring migration phases, both exhibiting similar microbial profiles enriched in *Proteobacteria* and *Planctomycetota*; and (D) autumn migration, with *Planctomycetota* as the dominant phylum, followed by *Proteobacteria* and *Verrucomicrobiota*.

Microbial composition in migratory birds fluctuates across phases, shaped by diet and habitat (Fig. 1A and B, Fig. 2). Migration alters nutritional intake and toxin exposure, influenced by interspecies variations in gut morphology [32–34]. These shifts can impact migratory success [35]. We incorporated results from published β-diversity analyses showing significant phase-dependent turnover in community composition [26, 36].

Metagenomic studies show that migratory-phase communities often exhibit lower modularity and co-occurrence, reflecting instability from dietary transitions and physiological trade-offs [24, 37]. Such reductions in microbial modularity indicate a breakdown of stable ecological interactions and reduced network stability [38]. This instability likely arises because rapid dietary shifts, elevated energetic demands, and altered gut transit during migration disrupt stable mutualistic associations among taxa [24, 39], resulting in more fragmented and weakly connected microbial networks [40]. We summarize studies detailing species-specific compositional shifts across migratory phases (Table S1) and find that avian gut microbial communities are not static but instead undergo rapid, phase-linked restructuring driven by location-specific diet and environmental exposure [41].

### Convergence with local microbiota at stopovers

Migrants often begin to acquire microbiota resembling resident birds within the first 24–48 h at stopover sites [26], although the pace and degree of alignment vary across species and habitats. This microbial shift is likely facilitated by shared foraging habits and exposure to local soil, water, and dietary inputs, underscoring the plasticity of the avian microbiota and its sensitivity to environmental conditions. This phenomenon involves not only taxonomic shifts but also functional reconfigurations, as migratory birds adopt microbial taxa with metabolic functions tailored to local ecological conditions [24]. Such convergence suggests that environmental filtering at stopovers exerts a dominant influence on gut microbiota composition, potentially facilitating rapid physiological acclimatization to transient ecological conditions. Beyond taxonomic turnover, migrants also adopt microbial groups with metabolic functions tailored to local ecological conditions [24]. Across multiple species, studies report that stopover habitats may act as environmental filters that rapidly draw migrant microbiota toward resident-like community profiles [20, 26, 27, 42]. However, the speed and extent of convergence vary among species and habitats, reflecting differences in diet, stopover ecology, and sampling resolution across studies (Table S2).

### Comparative insights: migrants versus residents

Migratory and resident birds often exhibit distinct gut microbiota due to differences in diet, evolutionary history, and geographic ranges [36, 43, 44]. Migrants undergo rapid dietary shifts and encounter diverse environmental microbial pools along flyways, contributing to higher temporal variability and frequent microbial turnover [24, 26, 28]. In contrast, resident birds typically forage in stable habitats, leading to more consistent microbial exposures [36, 45]. Host phylogeny further shapes baseline microbiota structure, with stronger phylogenetic filtering often observed in resident species relative to migrants [43, 44].

Comparative studies have assessed microbiota divergence by examining differences during migration [46] or after the migration period [47], and between migratory and resident populations [26]. Comparisons show that migration drives microbial restructuring, impacting immunity and energy regulation (Table S3). Migrants often show elevated *Proteobacteria* richness, which includes opportunists associated with inflammation and dysbiosis [48, 49]. Network-level disruption at stopovers has been reported, with reduced modularity potentially increasing susceptibility to pathogen establishment [50, 51].

## Factors shaping the gut microbiota during avian migration

Early-life microbial development provides the baseline from which later migratory shifts occur [45]. Migratory birds endure a cascade of physiological and environmental challenges during their annual journeys, ranging from internal hormonal fluxes to external ecological pressures that can trigger dynamic remodeling of the gut microbiota [52]. This process is influenced by stress-induced changes [53, 54], including behavioral disruptions, environmental exposures (altitude, heat, pharmaceutical residues, encounter with novel pathogens, and ingestion of environmental pollutants) [55], dietary transitions [56], developmental immaturity [56], and long-term host–microbiota co-adaptation [57]. The gut microbiota, when shifted from its balanced state of eubiosis, may transition into dysbiosis, favoring opportunistic pathogen proliferation [58]. Stressors such as heat, nutrient overload, and immune suppression can precipitate this disruption, promoting the overgrowth of pathogenic bacteria like *Clostridium perfringensi* [59, 60]. The resulting inflammation, epithelial damage, and impaired nutrient assimilation not only favor pathogen expansion but may also compromise migratory performance [61, 62].

Dysbiosis tends to be more pronounced in immature birds because their gut microbiota is still developing. At this stage, the community shows high temporal variability and has not yet reached the stable, adult-like structure reported in poultry and avian studies [63, 64], making it more vulnerable to disturbance. High-protein and high-fat diets can favor pathogen growth in the cecum, with retrograde migration into the ileum and jejunum via retroperistalsis, triggering toxemia and gut dysfunction [65, 66]. Understanding these interactions is essential, as microbial shifts are not incidental byproducts of migration but integral to how birds manage energy allocation, immune defense, and ecological adaptability enroute.

### Early-life ontogenetic shifts in gut microbiota and their impact on migration readiness

Avian gut microbiota evolves dynamically through distinct life stages, underpinning critical physiological and ecological processes [56]. Among these, migration represents one of the most energy-intensive transitions, and microbial maturation is closely linked to migration readiness. From early colonization post-hatching to adult metabolic fine-tuning, to genetic microbial shifts equip birds with adaptive capabilities essential for successful migration.

Microbial seeding begins shortly after hatching [67], although some evidence suggests that limited exposure may occur prenatally [68]. Nest environment and parental inputs drive a rapid rise in gut bacterial abundance within a few days [68, 69], with vertical transmission via cloacal or oviductal routes [70–73]. However, in shorebirds early microbiota assembly appeared largely shaped by environmental factors rather than host phylogeny [36]. Moreover, diet-mediated maturation of gut flora was observed in chicks of Japanese rock ptarmigan (*Lagopus muta*), where early consumption of adult-like diets accelerated cecal colonization with enhanced nutrient assimilation necessary for growth [74]; this early microbial establishment is critical for growth and the subsequent energetic demands of migration.

Environmental factors further shape early microbial communities. In the great tit (*Parus major*), nesting environment strongly shaped gut microbiota, at times overriding genetic effects [75], while convergence patterns observed in woodlark (*Lullula arborea*) and Eurasian skylark (*Alauda arvensis*) chicks raised in shared habitats further confirm environmental filtering [76]. Yet, genetic contributions persist: in a cross-nesting experiment, great spotted cuckoo (*Clamator glandarius*) and Eurasian magpie (*Pica pica*) nestlings reared together showed convergence in esophageal microbiota but retained species-specific cloacal profiles, reflecting genetic influence on deeper gut compartments [77].

Developmental trajectories ranging from altricial to precocial also influence the microbiota establishment [36]. In altricial chicks, e.g. zebra finch (*Taeniopygia guttata*), the microbiota establishment is rather gradual, and microbial transmission occurs indirectly through parental fecal and cloacal materials deposited in the nest [78, 79]. Early communities dominated by *Proteobacteria* transition to *Firmicutes*-rich profiles within 3 weeks post-hatching [69, 80], promoting energy efficiency and weight gain [75, 81].

Conversely, precocial shorebirds such as the dunlin (*Calidris alpina*) and semipalmated sandpiper (*Calidris pusilla*) exhibit rapid microbial succession within 2 days due to early foraging exposure with dominance shifting toward *Clostridia* and *Gammaproteobacteria* [68]. Adding further depth, research on the Eurasian kestrel (*Falco tinnunculus*) [69] and American kestrel (*Falco sparverius*) [82] reveals that nestlings harbor higher microbial diversity, particularly enriched in *Firmicutes* and *Actinobacteria*, compared to adults [69, 83]. These bacterial groups are associated with enhanced energy storage and metabolic regulation [84], supporting fat accumulation necessary for bird growth [85]. This suggests that early-life microbial configurations may be critical for future anatomical, physiological, and metabolic traits [82].

In summary, ontogenetic development of gut microbiota is intricately linked with migratory preparation and performance. From early-life microbial seeding to adult-stage functional refinements, the plasticity of the gut ecosystem potentially primes birds to meet the physiological, immunological, and ecological challenges of migration.

### Stress-response mechanism

**Figure 3:**
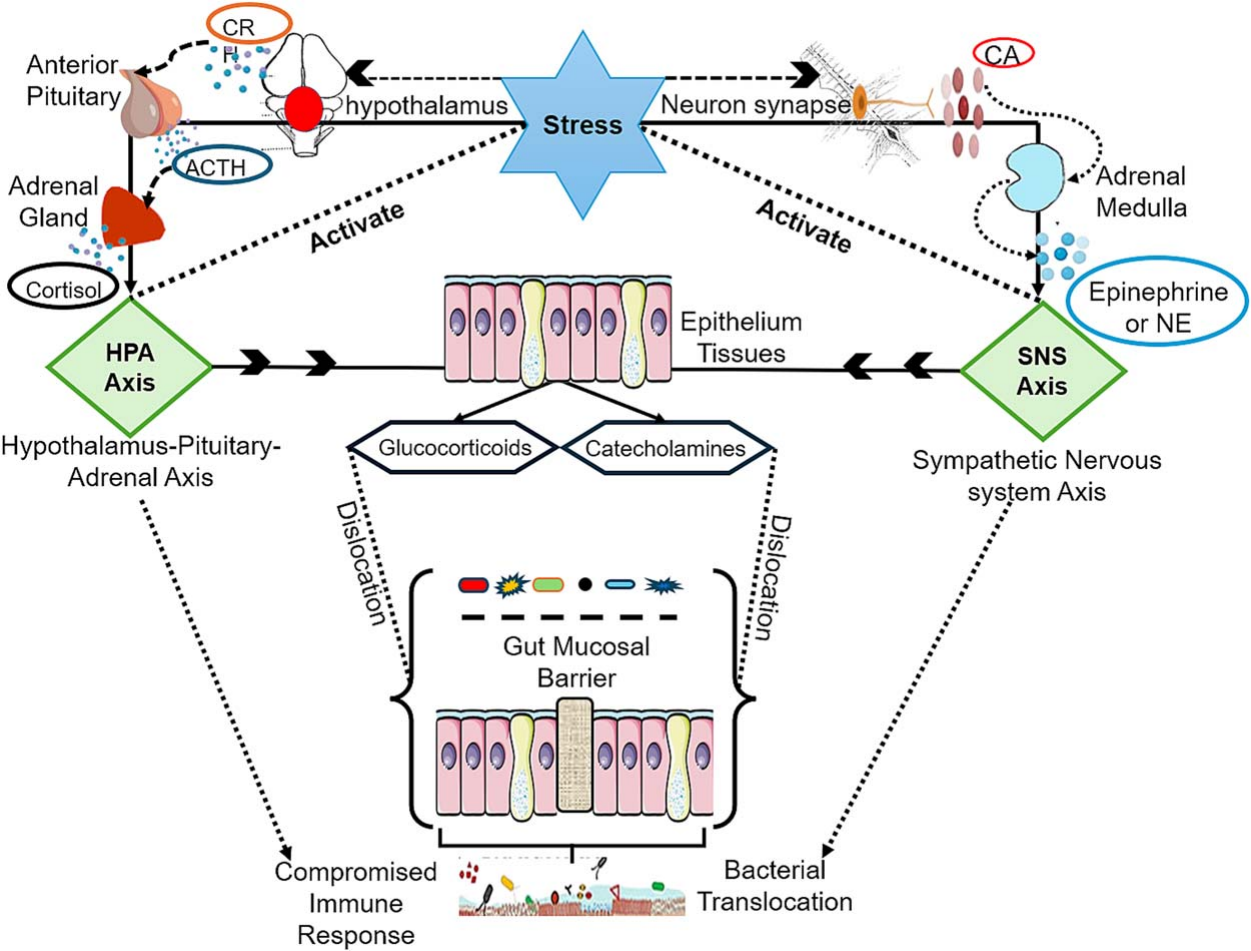
Stress-induced disruption of the gut mucosal barrier. The conceptual figure was reproduced from the data obtained by the following sources: [241–243]. The figure shows how stress disrupts the gut mucosal barrier through the hypothalamus–pituitary–adrenal (HPA) axis and the sympathetic nervous system (SNS) axis. Stress activates the hypothalamus, leading to the release of ACTH from the anterior pituitary and cortisol from the adrenal gland (HPA axis). Simultaneously, the SNS axis releases catecholamines (epinephrine and norepinephrine). These stress hormones cause the dissociation of the gut mucosal barrier, leading to compromised immune response and bacterial translocation.

The biological stress response in migratory birds is orchestrated primarily by the hypothalamic–pituitary–adrenal (HPA) axis and the sympathetic nervous system (SNS). Their activation during migration triggers the release of glucocorticoids (e.g. corticosterone), catecholamines, and neuropeptides, which collectively influence immune regulation, gastrointestinal physiology, and microbial dynamics [86–88] (Fig. 3). While these responses are evolutionarily adaptive and aim to maintain homeostasis, chronic or intense stress, common during endurance flights, extreme temperatures, or predator exposure can lead to dysregulation and gut microbiota alterations [89].

Stress hormones influence the gut via multiple overlapping pathways [90]. Catecholamines and glucocorticoids alter the intestinal environment by modulating epithelial integrity, mucosal immunity, and secretory function [91, 92]. Neuroendocrine signals may directly influence microbial growth and virulence gene expression [92]. In parallel, stress disrupts vagal tone and enteric nervous system signaling, reducing peristalsis and altering gastric secretions, changes that affect gut transit time and nutrient availability, both of which are key determinants to microbiota structure [86].

In migratory contexts, stress-induced ischemia–reperfusion cycles from sustained physical exertion may reduce intestinal blood flow, increasing gut permeability. This phenomenon commonly referred to as “leaky gut” may facilitate the translocation of bacteria or microbial products into the bloodstream, thereby exacerbating microbial imbalance [93]. Simultaneously, glucocorticoid-driven immunosuppression impairs gut-associated lymphoid tissue, weakening local immune surveillance and enabling opportunistic taxa to expand [94]. Together, these interactions highlight stress as both a physiological regulator and microbial disruptor. For migratory birds, the capacity to buffer stress without inducing dysbiosis may be key to sustaining migratory performance.

### Barrier breakdown: physiological strain and gut vulnerability

Beyond systemic stress, migration imposes direct mechanical and nutritional strain on the gut, leading to altered morphology, compromised epithelial barriers, and reduced mucosal immunity collectively referred as “gut barrier breakdown” [95]. This state fosters instability in microbiota composition, allowing opportunistic taxa to thrive. Prolonged flight and energy depletion may cause gut atrophy, increased permeability, and weakened immune surveillance, reducing host control over microbial communities [96]. In *Calidris* shorebirds, migration-induced strain favored specific taxa (e.g. *Corynebacterium*) without collapsing microbial structure [46], suggesting adaptive remodeling. In contrast, steppe buzzards in poor condition exhibited reduced α-diversity, linking gut integrity to host fitness [30].

Experimental models support these patterns. Birds implanted with corticosterone to mimic chronic stress showed decreased microbial diversity [29]. Similarly, infection with avian influenza virus increased glucocorticoid levels and disrupted barrier integrity, with corresponding microbial shifts [97]. Migrating red knots (*Calidris canutus*) and ruddy turnstones (*Arenaria interpres*) have been found to carry high loads of potential pathogens (e.g. *Campylobacter* spp.) during spring migration, likely due to mucosal barrier weakening under physiological strain [98]. However, many species appear to preserve a resilient core microbiota [99, 100], potentially aiding in pathogen exclusion and post-migratory recovery. However, this resilience hypothesis requires broader testing to determine whether it represents a widespread adaptation or varies by species, ecology, or pathogen exposure.

### Circadian rhythms and microbial oscillations during migration

Circadian rhythms (~24-h cycles in physiology and behavior) are key regulators of host–microbiota interactions [101, 102]. In migratory birds, these rhythms are frequently disrupted due to shifting photoperiods, irregular feeding, and altered foraging across latitudes. Nocturnal flights and prolonged stopovers further desynchronize internal clocks, potentially undermining gut homeostasis [103].

Experimental studies in poultry offer relevant analogs. Chickens exposed to extended light cycles exhibited altered gut microbial composition and impaired colonization, likely mediated through disrupted circadian gene expression [104]. Such misalignment also reduced short-chain fatty acid (SCFA) production, compromised immune function, and increased gut permeability—hallmarks of barrier dysfunction [102]. Microbial taxa also exhibit circadian oscillations, responding to host feeding schedules and melatonin cycles [105]. Disruption of these microbial rhythms has been shown to impair immune regulation and increase systemic inflammation in mammals and poultry [101, 106].

Although direct data on migratory birds remain limited, analog evidence suggests that circadian disruption could reduce microbial diversity, induce leaky gut, and impair metabolic resilience [101, 102]. Comparative studies of diurnal versus nocturnal migrants and longitudinal microbiota tracking would clarify how circadian control supports gut health during migration.

### Environmental extremes: microbial resilience and stress responses

The gut microbiota’s resilience to stress-induced perturbations is critical for maintaining host homeostasis during migration. Migratory birds face unpredictable environmental extremes; understanding how these stressors affect microbial stability is essential for addressing physiological and ecological challenges.

#### Altitude hypoxia and microbial metabolic shifts

High-altitude environments (≥2500 m) impose hypobaric hypoxia, reducing oxygen delivery to peripheral tissues and triggering oxidative stress, inflammation, and impaired gastrointestinal motility [107, 108]. These stressors disrupt gut microbial composition both through hypoxia-driven changes in intestinal oxygen availability and through downstream effects on host metabolism and immune activation [109–111].

Altitude imposes selective pressure favoring taxa adapted for oxidative stress resistance, nitrogen metabolism, and fermentative pathways [112, 113]. Evidence from animal models shows that gut microbiota facilitates adaptation to hypoxia by enhancing glucose metabolism. Key microbial groups, such as lactic acid bacteria and *Streptococci*, degrade non-starch polysaccharides and produce SCFAs, which modulate insulin sensitivity, fat, glucose metabolism, host energy balance, and thermogenesis via GPR43 signaling [114–117]. A compelling example comes from highland Eurasian tree sparrows (*Passer montanus*), which exhibit enlarged digestive organs, longer intestinal villi, a shift toward protein-rich diets, and a restructured gut microbiota compared to lowland conspecifics. Liver metabolomics point to enhanced metabolic efficiency likely shaped by host–microbe interactions, underscoring the microbiota’s contribution to hypoxia tolerance [118].

In summary, the gut microbiota may act as a metabolic buffer and adaptive partner during high-altitude migration. Its functional plasticity, particularly in energy metabolism and nutrient assimilation, might help birds meet the dual demands of sustained flight and oxygen scarcity, suggesting a key symbiotic axis in extreme environmental resilience. However, further experimental validation is needed to substantiate these functional roles in migratory birds.

#### Thermal stress and gut ecosystem collapse

Thermal stress can structurally and functionally impair avian intestines, with most mechanistic insights derived from poultry models [119–122]. Even short heat exposure (4–6 h) can cause villus atrophy, crypt shortening, epithelial shedding, and impaired nutrient absorption [123]. Tight junction protein disruption increases gut permeability, facilitating bacterial endotoxins to translocate into circulation and triggering inflammation and systemic immune responses [122, 124]. Heat stress also shifts microbial communities, lowering diversity and reducing beneficial taxa like *Lactobacillus* and *Bifidobacterium*, which diminishes SCFA production and compromises barrier function [119, 120]. Interestingly, gut microbiota may aid thermal adaptation. In mice, extreme temperature exposure led to distinct microbial configurations. Microbiota transplants from heat-stressed donors conferred thermal tolerance by modulating insulin signaling and energy metabolism [125], indicating a microbiota role in thermoregulation. In wild birds, eastern bluebird (*Sialia sialis*) nestlings showed heat-induced microbiota shifts, unlike thermally stable tree swallows (*Tachycineta bicolor*) [126], suggesting species-specific microbial resilience.

Poultry models show that heat alters the *Firmicutes*-to-*Bacteroidetes* ratio and increases pathogen susceptibility [127, 128]. If mirrored in migratory birds, this could impair barrier function and nutrient absorption. Migrants frequent stopovers in arid, tropical, or urban environments where daytime temperatures exceed 40°C [129], e.g. passerines stopping in the Negev Desert or African Sahel [130], conditions comparable to those used in poultry heat-stress studies. While behavioral and physiological thermoregulatory mechanisms are well documented, the susceptibility of gut microbiota to thermal extremes remains largely unexplored. Although evidence is accumulating that heat stress affects migratory bird physiology, its implications for gut microbial structure and function represent a critical, yet understudied, frontier. To bridge this gap, integrative studies incorporating *in situ* temperature tracking, microbiota profiling, and gut integrity biomarkers are urgently needed. Understanding thermal impacts on the avian gut microbiota is vital in the face of climate change, which threatens to intensify temperature extremes along migratory routes.

#### Toxin-mediated gut microbiota remodeling and adaptive responses

Toxic exposures, including pollutants and toxins such as heavy metals, pesticides, and microplastics [131], are increasingly recognized as significant environmental stressors that can disrupt the gut microbiota [132] of wildlife [133]. Notably, gut microbiota plays a critical role in pathogen defense and facilitates detoxification [134, 135], and its disruptions can compromise host health and fitness. Recent findings from migratory songbirds and urban passerines suggest heightened microbiota vulnerabilities when navigating through anthropogenic landscapes [136].

Microplastics (MPs) including polyethylene, polypropylene, and polystyrene (PS) impair nutrient absorption and antioxidant defense mechanisms in birds [137, 138]. Across avian models, MP exposure consistently induced dysbiosis [139]. For instance, PS exposure in chickens inhibited lipid metabolism and altered the *Firmicutes*-to-*Bacteroidetes* ratio [138, 140, 141], while in Muscovy ducks it increased pro-inflammatory genera like *Streptococcus* and *Helicobacter* [142]. MP exposure was also found to reduce beneficial taxa (*Lactococcus, Bifidobacterium, Butyricicoccus*) and enriched potential pathogens (*Enterococcus, Turicibacter*) [138]. Co-exposure to PS-MPs is marked by an increase in antibiotic resistance genes and loss of commensals, which fosters colonization by zoonotic or plastic-degrading bacteria [142, 143], ultimately compromising digestion, immunity, and host resilience [144].

Heavy metals such as cadmium (Cd), lead (Pb), and copper (Cu) similarly destabilize gut microbial communities. In tree sparrow nestlings, metal exposure reduced alpha diversity and enriched pathogens (*Aeromonas, Pseudomonas*), while it suppressed the beneficial genera *Ruminococcus*. Interestingly, *Lactobacillus*, a known probiotic with detoxification potential, was enriched, suggesting both vulnerability and adaptive microbial responses. Functional analysis revealed downregulated digestive pathways and growth-related functions. Conversely, energy metabolism and antioxidant pathways, such as glutathione metabolism, were upregulated and pathways were linked to DNA repair, antibiotic synthesis, and immune responses, reflecting a microbiota-driven defense response [145].

Similarly, pesticide exposure also remodels gut communities in wild birds. In Montagu’s harrier (*Circus pygargus*) nestlings, higher pesticide loads correlated with increased *Bacteroidota* and *Leifsonia* phyla linked to xenobiotic degradation alongside enrichment of pathogenic *Pseudomonas* and *Stenotrophomonas*. Notably, *Burkholderia*, a genus involved in pesticide breakdown, was more abundant in less-contaminated individuals [146], suggesting a protective role. Japanese quails (*Coturnix japonica*) exposed to trichlorfon, an organophosphate insecticide, showed increased *Proteobacteria*, a dysbiosis marker linked to inflammation in mammals [49, 147, 148].

Collectively, environmental toxins drive both detrimental and adaptive microbial shifts in wild birds. For migratory species, especially those foraging in intensively farmed or polluted habitats, such remodeling could affect not only health and migratory performance but also zoonotic spillover risk.

### Diet-induced microbial remodeling

The gut microbiota of migratory birds exhibits remarkable plasticity in response to dietary shifts, reflecting both immediate nutritional inputs and broader ecological adaptations. While host genetics and phylogeny play a foundational role, diet is often the dominant force, influencing both composition and function. In barn swallows (*Hirundo rustica*), fine-scale seasonal variation in insect prey (e.g. *Sarcophaga, Helina*) corresponds with shifts in core gut taxa such as *Enterococcus* and insect symbionts like *Rickettsia*. These fluctuations are accompanied by functional changes in nucleic acid and sugar metabolism, underscoring tight diet–microbiota coupling [149].

Similarly, black-necked cranes (*Grus nigricollis*) exhibited seasonal microbial remodeling aligned with dietary transitions from animal (e.g. insects and small vertebrates) to plant sources. Fiber- and phenylpropanoid-degrading taxa (e.g. *Lactobacillaceae, Eggerthellaceae*) increased during plant-rich periods, supporting detoxification and energy extraction [150]. A broader survey at Poyang Lake found that dietary niche rather than host phylogeny primarily structured gut microbial communities across herbivorous, omnivorous, and carnivorous migratory birds. Omnivores harbored the most diverse microbiota; functional gene profiles reflected niche-specific processes such as fiber degradation in herbivores and glycan metabolism in omnivores [51].

Dietary experiments in great tits (*P. major*) showed rapid restructuring: seed diets enriched fiber-fermenters (e.g. *Blautia, Ruminococcus*), while mealworm diets favored protein-associated taxa like *Akkermansia* and *Romboutsia* [151]. Notably, microbial communities only partially reverted upon returning to an original diet, suggesting lasting dietary imprints on microbiota [152]. This trade-off between flexibility and vulnerability is evident in the critically endangered Siberian crane (*Grus leucogeranus*). Seasonal shifts from high-fiber wetland plants to carbohydrate-rich crops like rice and lotus rhizomes displayed increased microbial diversity and enhanced carbohydrate metabolism, driven by taxa such as *Lactobacillaceae*. However, this dietary switch also coincided with a proliferation of potential pathogens (*Enterobacteriaceae, Streptococcaceae*), likely due to shared foraging grounds with domestic poultry [153].

Similarly, in sympatric overwintering great bustards (*Otis tarda*) and common cranes (*Grus grus*), diet-specific microbial differentiation persisted despite interspecies variation. For example, peanut-fed cranes showed elevated lipid metabolism, while wheat-fed birds favored glycan biosynthesis [154], revealing how macronutrient composition fine-tunes gut function. Collectively, these studies converge on a key insight: migratory bird gut microbiota is not only shaped by what birds eat but how consistently and in what ecological context. Bird microbiota responds swiftly to dietary input, yet full recovery post-shift may be limited. Moreover, exposure to anthropogenic diets may increase pathogen load, with consequences for health and population viability. Understanding these microbiota–diet dynamics is crucial for conservation efforts, particularly when assessing habitat quality, guiding feeding interventions, and forecasting migratory resilience in a rapidly changing world.

### Host–microbiota co-evolution and environmental filtering

The avian gut microbiota not only reflects ecological fluctuations but is also increasingly recognized as an integral component of host evolutionary architecture [155, 156]. Host–microbiota co-evolution involves the reciprocal selective pressures between birds and their gut symbionts, whereby gut microbial assemblages become evolutionarily fine-tuned [157]. Experimental studies in selectively bred chickens have shown that alterations in host humoral immunity and energy metabolism drive stable, heritable shifts in gut microbiota, effectively forming a functional holobiont [158, 159]. These findings suggest that host genetics may shape microbiota architecture, particularly traits linked to immune regulation and energy storage. Evidence for symbiosis between birds and their gut microbiota remains limited, partly due to evolutionary reductions in digestive tract size—a consequence of flight-related adaptations [160].

Phylosymbiosis, where gut microbial community structure reflects host phylogeny, offers compelling evidence for co-evolution and has been observed in several avian clades [155]. For example, closely related cranes such as the black-necked crane (*G. nigricollis*) and common crane (*G. grus*) share highly similar gut microbial profiles, suggesting conserved host–microbe relationships [161]. Similarly, in 15 species of wood warblers (*Phylloscopus sibilatrix*), microbiota structure aligned more closely with evolutionary relatedness than with dietary variation [162]. These patterns mirror findings in non-avian taxa and support the idea that host lineage can shape microbial trajectories across deep evolutionary time [156].

However, phylogeny alone does not dictate microbial assembly. Ecological variables, particularly diet and habitat, can override phylogenetic constraints, driving convergence among distantly related species. A striking example involves the blood-feeding vampire ground finch (*Geospiza septentrionalis*), which shares core microbial functions with the vampire bat (*Desmodus rotundus*), despite vast evolutionary distances [163]. Similarly, Galápagos finches and sympatric flycatchers show microbial convergence based on shared foraging strategies rather than ancestry [162, 164]. Beyond taxonomic convergence, emerging data reveal molecular-scale host–microbiota co-adaptation. In the domestic Sichuan white goose, parallel genome and metagenome analyses showed expansion of host genes for energy and carbohydrate metabolism, accompanied by functional enhancements in gut microbes, an elegant example of co-evolution driven by dietary specialization [165]. This supports the holobiont paradigm, where host and microbiota co-evolve as an integrated unit, jointly optimized for environmental and physiological demands.

While most evidence derives from domestic or non-migratory birds, these evolutionary dynamics likely underpin microbiota plasticity in migratory species. Migration imposes extreme stress, requiring microbiota that are both stable and adaptable. Thus, long-term co-evolution may confer resilience while environmental filtering ensures functional flexibility, a dual mechanism supporting health and performance during migration.

## Functional adaptations: gut microbiota as physiological allies

The gut microbiota is increasingly regarded as a metabolically active and immunologically engaged “organ,” contributing to energy regulation, nutrient absorption, vitamin biosynthesis, immune development, and gut maturation [166–169]. These functions become particularly critical for migratory birds, whose annual journeys demand substantial physiological remodeling, including fueling, detoxification, immune adaptability, and gastrointestinal plasticity [24, 56].

While much of our understanding of gut microbiota functions originates from poultry models, these provide mechanistic analogs for migratory birds. In poultry, gut microbes have been observed to facilitate digestion [170], synthesize bioactive molecules for the host [171], and interact with the immune system [94]. Emerging studies in migratory birds suggest similar roles, which face extreme physiological states such as hyperphagia, prolonged fasting, and prolonged exertion. Under these conditions, microbiota may enhance metabolic flexibility, support fat storage, and modulate immune responses to maintain homeostasis [168, 172].

### Microbial mediation of energy demands and fat storage

**Figure 4:**
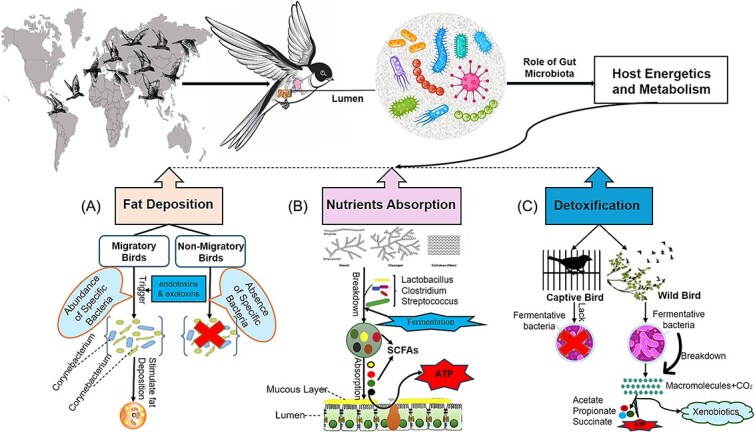
Gut microbiota in shaping the host energetics and metabolism. The data for the figure were sourced from the following sources: 3(a) [4, 190, 191], 3(b) [170, 171], 3(c) [175]. The conceptual figure provides a visual illustration of the role of gut microbiota in influencing host energetics and metabolism through fat deposition, nutrient absorption, and detoxification. (A) shows that migratory birds may have enhanced fat deposition mechanisms facilitated by their gut microbiota, aiding in energy storage necessary for long-distance flights. In contrast, non-migratory birds may not have these mechanisms. (B) Highlights how specific gut bacteria such as *Lactobacillus, Clostridia*, and *Streptococcus* aid in nutrient absorption by fermenting dietary fibers into short-chain fatty acids (SCFAs), which are then absorbed by the host to generate ATP and support energy metabolism. (C) Demonstrates the detoxification process where fermentative bacteria in wild birds break down xenobiotics and environmental toxins into less harmful compounds like acetate, propionate, and succinate, thereby maintaining metabolic health while captive birds lack these bacteria.

The gut microbiota plays a pivotal role in meeting the intense energy demands of migration [5]. Its dynamic composition allows birds to adapt to changing diets and metabolic needs [173, 174]. For example, the wild Western capercaillie (*Tetrao urogallus*) meets its winter energy requirements by consuming resinous coniferous needles, which reduces the diversity of its cecal bacterial community compared to more diverse diets [175], while the captive capercaillie birds lack certain fermentative bacterial species, such as those from the *Synegistes* phylum, which are essential for fermenting carbohydrates into acetate, propionate, and succinate (SCFAs), thereby contributing to energy supply and detoxification [175]. The divergence in gut microbiota between wild and captive capercaillie suggests that limited movement and reduced ecological interactions in captivity may constrain microbial diversity (see Fig. 4C).

Migratory birds rapidly accumulate fat to fuel endurance flights while avoiding complications such as inflammation or liver damage [176], relying primarily on lipid metabolism, although protein and carbohydrate metabolism also contributes. Gut microbes support this by enhancing lipid absorption, producing signaling molecules, and modulating host metabolism [177–179]. These regulatory roles, well documented in hibernators, may similarly apply to migratory birds facilitating pre-migratory fat accumulation, protein balance through nitrogen recycling, and inflammation control [174, 180, 181]. The gut microbiota plays a pivotal role in energy homeostasis by producing SCFAs, which regulate insulin sensitivity, adiposity, gluconeogenesis, and satiety [75, 182, 183], while branched-chain amino acids contribute to energy metabolism [184] during the hypermetabolic state of migration.

Emerging metagenomic evidence supports the important roles of gut microbiota. In ruddy turnstones (*A. interpres*), microbial genes involved in polyunsaturated fatty acid (PUFA) metabolism correlate with pre-migratory weight gain [25]. Since PUFAs efficiently fuel migration [185], microbial restructuring may support energetic readiness. Similarly, in blackpoll warblers (*Setophaga striata*), microbial pathways for vitamin, amino acid, and fatty acid biosynthesis during migratory stopover periods were associated with subcutaneous fat stores and body mass [28] in migratory birds.

Additionally, gut microbes enhance nutrient extraction vital for high-energy diets rich in fruits or insects. Gut microbiota decomposes complex carbohydrates and polysaccharides, enhancing nutrient absorption and metabolism [3] (Fig. 4C). In poultry, a clear link between gut bacteria and host growth has been observed, with bacteria such as *Lactobacillus, Clostridium*, and *Streptococcus* playing significant roles in the degradation of non-starch polysaccharides and the synthesis of crucial molecules like SCFAs [170, 171]. These SCFAs offer readily available energy during flight with limited food intake and reduce intestinal pH, preventing pathogen colonization (see Fig. 4B), while also promoting the differentiation and expansion of T cells into T regulatory cells by binding to receptors such as Toll-like receptors and G protein–coupled receptors on immune cells [98, 186–188]. These roles of SCFAs highlight their abundance and importance in microbial degradation during the high-energy demands of migration [18, 189].

Evidence from migratory buntings supports the metabolic role of SCFAs, with elevated levels of butyrate, lactic acid, and L-valine in the blood serum during migration [184]. These metabolites are rapidly transported to muscles from liver fat during endurance flight, supported by high expression of FAT/CD36 and fatty acid binding protein gene expression in muscle [176]. Conversely, some bacteria may promote fat storage through inflammation. *Corynebacterium*, for instance, may trigger endotoxin-mediated inflammation that enhances energy harvest and fat storage [4, 190, 191] (Fig. 4A). In experimental mice, Gram-negative bacteria increase fat deposition via endotoxins [192–194]. Such mechanisms might explain the high prevalence of pathogenic bacteria in migrating birds, such as *Corynebacterium* and *Campylobacter* in American shorebirds [98] and *Escherichia* and *Paracoccus* in passerines [195]. However, experimental validation of these mechanisms in migratory avian models remains an important area for future research.

### Immune modulation and gut microbiota in host defense

Migratory birds encounter numerous pathogens during their extensive journeys, necessitating strong immune defenses. The gut microbiota serves as a key modulator of these defenses, shaping immune responses by promoting stability and reducing susceptibility to infection [194, 196]. These microbes combat pathogens both directly, through competitive exclusion, and indirectly, by activating host immune pathways. Commensal microbes also produce antimicrobial compounds [94]; for instance, *Lactobacillus* and *Bifidobacterium* are linked to resistance against gastrointestinal infections by producing lactic acid and other substances that reduce gut pH and inhibit pathogens like *Salmonella* and *Clostridium* [197, 198].

**Figure 5:**
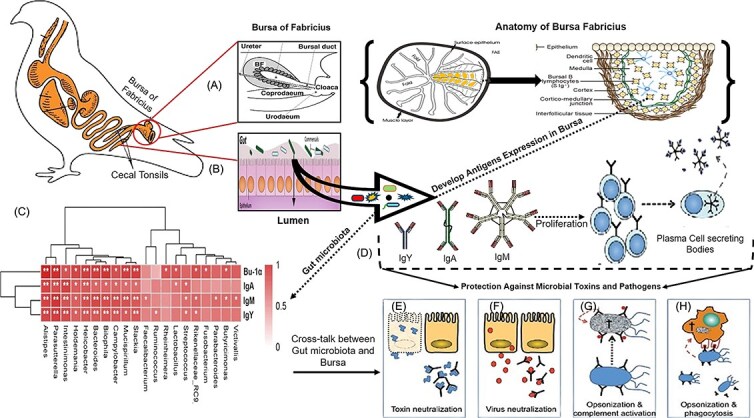
Cross-talk between the bursa of Fabricius and gut microbiota for the B-cell development and antibody production. The figure is modified with the permission: 4a [204]. Copyright 2024, Elsevier. 4b [244]. Copyright 2024, Elsevier. 4c [205], 4d, e, f, g, h. copyright 2024, the poultry site, [245] author permission. The conceptual figure illustrates the interaction between the bursa of Fabricius and gut microbiota in birds, which is crucial for B-cell development and antibody production. (A) Details the bursa’s anatomy, while (B) shows the cecal tonsils and (C) presents a heatmap correlating gut microbiota with antibody types (IgY, IgA, IgM), highlighting microbial influence on immune responses. (D) to (H) demonstrate how antigens from the gut lumen lead to B-cell proliferation and antibody production, which then neutralize toxins and pathogens through various mechanisms.

In wild ducks, shifts in gut microbiota composition correlate with increased susceptibility to influenza virus [199], underscoring the importance of microbial stability for antiviral defense. Similarly, in ostriches, pathogen-induced dysbiosis impairs immunity and elevates infection risk [200, 201]. These patterns suggest that microbial stability is essential for disease resistance during migration. In addition to innate pathogen defense, the gut microbiota also influences adaptive immunity through its interaction with lymphoid organs such as the bursa of Fabricius. This organ, located in the cloaca, is the primary site for B-cell development and hematopoiesis in birds [6]. The bursa is colonized by microbes shortly after hatching, which promotes the proliferation and maturation of bursal B cells [202, 203] (Fig. 5A). Experimental ligation of the bursal duct before hatching results in reduced natural antibody production in chickens, suggesting systemic immunomodulatory roles for gut microbes [204]. While it is established that gut microbiota influences B-cell maturation, the extent to which these interactions shape migratory immune phenotypes remains underexplored. While gut microbiota may not directly influence the shape and size of the bursa of Fabricius, it significantly influences proportions of B-cell production (Fig. 5B, D, E, F, G, and H). Bursa-dependent B cells are crucial for antibody production in birds [204]. Moreover, the development of the expression of Bu-1a (a marker for B-cell antigen), immunoglobulin A (IgA), immunoglobulin M (IgM), and immunoglobulin Y (IgY) in the bursa of Fabricius is associated with the presence of specific gut microbes. It was observed that at the phylum level, *Bacteroidetes* and *Deferribacteres* were positively associated with Bu-1a, IgA, IgY, and IgM expression. At the genus level, *Intestinimonas, Bilophila, Parasutterella, Bacteroides, Helicobacter, Campylobacter*, and *Mucispirillum* showed positive correlations with Bu-1a, IgA, IgY, and IgM [205] (Fig. 5C).

Emerging evidence also highlights that microbial components such as lipopolysaccharide, flagellin, and microbial DNA can stimulate broad-spectrum antibody production, potentially enhancing immune readiness during pathogen-rich migratory periods [206, 207]. Furthermore, diet-driven microbial restructuring at migratory stopovers may further modulate immune dynamics. For instance, *Escherichia coli* has been linked to protection against avian malaria, while high levels of *Bacteroides* have been associated with increased susceptibility [208–210]. Thus, the presence of certain Gram-negative taxa may reflect an evolved immune strategy rather than mere dysbiosis [20].

Despite growing evidence of the gut microbiota’s involvement in immune modulation, the mechanistic links during migratory stress remain unclear. Future studies using controlled manipulations such as antibiotic-induced microbiota depletion could clarify the roles of specific taxa in shaping B-cell maturation and antibody production during migratory stress.

## Global spillover of zoonotic risks and antimicrobial resistance via migratory birds

Migratory birds traverse continents, carrying diverse gut microbiota, including both commensals and pathogens. While these microbes serve essential physiological functions, migratory birds are increasingly recognized as vectors for zoonotic pathogens and AMR genes [211]. The wide-ranging migratory routes of these birds bring them into contact with a variety of environments, such as urbanized areas, agricultural zones, wetlands, and human settlements. However, bird-mediated transmission occurs within a broader system that also includes wastewater, livestock production, and human mobility and should therefore be viewed as one part of the wider AMR and pathogen landscape rather than a dominant driver. This diverse exposure increases the likelihood of microbial exchange between birds, livestock, and humans, thereby facilitating the spread of diseases and AMR genes [212, 213].

Birds are known reservoirs for several zoonotic pathogens, including *Salmonella* spp. [21, 214], *Campylobacter* spp. [215–217], *E. coli* [217], and avian influenza virus [218, 219]. Pathogen carriage in birds is influenced not only by migration but also by local ecological and anthropogenic factors, including habitat quality, environmental contamination, and proximity to livestock, which strongly shape the microbial communities birds acquire and transport. These microbes can infect both birds and humans, causing gastrointestinal, respiratory, and potentially pandemic illnesses (e.g. avian influenza) [220]. The widespread distribution of these pathogens is driven by the extensive migratory routes of birds, which carry them across continents, thus facilitating their global spread.

Migratory birds also contribute to the emergence and global spread of novel zoonotic agents. For instance, highly pathogenic avian influenza strains have spread via migratory waterfowl into poultry populations across regions [218, 219]. Similarly, *West Nile* has spread across continents, transmitted by infected birds [221, 222]. A large-scale Chinese study found that >30% of migratory bird fecal samples carried ≥14 human-associated pathogens and AMR genes (macrolide, quinolone, tetracycline, β-lactam), with loads between 10^5^ and 10^9^ copies/g [223]. USGS reports also show that migratory birds near human settlements often harbor antibiotic-resistant bacteria (usgs.gov).

In addition to pathogens, migratory birds are also important reservoirs for AMR genes, which confer resistance to common antibiotics used in human and veterinary medicine. AMR is a growing public health concern, and birds play a significant role in the dissemination of resistant bacteria. Migratory birds meet contaminated environments, such as sewage systems, agricultural areas, and landfills, which expose them to high levels of antibiotics and AMR bacteria [224–226]. Resistance has been observed to various antibiotic classes, including chloramphenicol, β-lactamase, quinolones, and macrolides. Early reports from Japan documented chloramphenicol-resistant *E. coli* strains in wild birds [227], and subsequent studies have detected resistance to methicillin (*Staphylococcus aureus*), vancomycin (*Enterococci, Salmonella* spp., *Vibrio cholerae*, and *Campylobacter* spp.), and carbapenems in avian isolates [216, 228–233].

**Figure 6:**
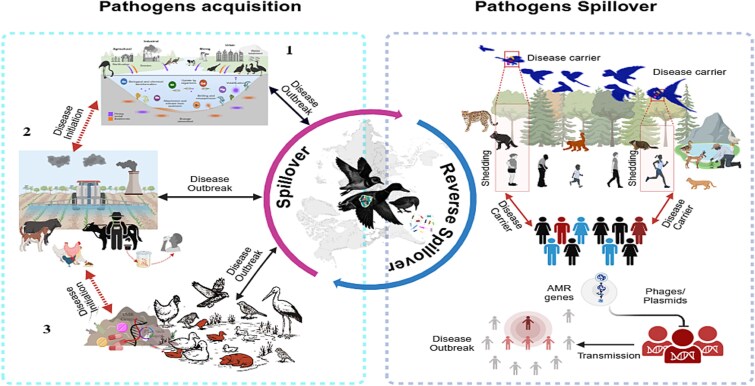
Mechanisms of zoonotic pathogens and antimicrobial resistance (AMR) spillover via migratory birds. This conceptual model outlines a three-phase pathway through which migratory birds facilitate the global dissemination of zoonotic pathogens and antimicrobial resistance genes. (1) Pathogen acquisition occurs as birds forage or dwell in contaminated environments, including agricultural runoff zones, urban discharge, and shared habitats with livestock, humans, and resident bird populations. During this stage, birds are exposed to polluted sediments, residual antibiotics, and heavy metals (e.g. Cd, Pb, Hg), promoting the uptake of bacteria, phages, and mobile genetic elements such as plasmids. (2) As carriers, birds serve as mobile reservoirs, transporting these microorganisms and resistance determinants across vast geographic distances. (3) In the spillover phase, pathogens and AMR genes are shed into new ecosystems via feces or direct contact, facilitating transmission to wildlife, domestic animals, and humans. The spillover process operates as a feedback loop: environmental contamination re-enters acquisition routes through sediment resuspension, biogeochemical transformation, and reintegration into ecological interfaces, thereby amplifying the risk of zoonotic emergence and resistance spread. The figure was created in https://BioRender.com.

Extended-spectrum β-lactamase (ESBL)-producing *Enterobacteriaceae*, such as *E. coli* strains carrying the CTX-M-15 and CTX-M-14 genes, have been widely reported in migratory birds [225]. Furthermore, plasmid-mediated AmpC β-lactamases, particularly CMY-2, which confer resistance to penicillin and cephalosporin, have also been detected in migratory species [225]. The mcr-1 gene, known for its role in colistin resistance, have been identified in migratory birds from diverse geographical regions, including European herring gulls (*Larus argentatus*) [226]. Recent genomic surveys have detected carbapenem-resistance genes (blaNDM, blaIMP-4) and tet (X4) in ESBL-producing *E. coli* (CTX-M-14/15) isolated from wild birds in Australia and Asia, often on conjugative plasmids (e.g. IncHI2-N) shared with human and livestock pathogens, evidence of active horizontal gene transfer [234–236]. The flocking behavior of migratory birds facilitates rapid microbial exchange within and across species [212, 213, 237, 238]. Intra- and interspecies interactions during migration, at breeding sites, stopovers, and urban foraging areas serve as hotspots for pathogen and AMR gene transmission [237, 238]. It enhances the potential for zoonotic spillover, particularly in regions with close human, livestock, and wildlife contact (Fig. 6).

Importantly, pathogen and AMR carriage in birds is strongly shaped by environmental exposure, land-use practices, and anthropogenic contamination, meaning migration is only one contributor to observed patterns. Implementing noninvasive methods, such as fecal sampling, during population monitoring offers valuable insights into pathogen carriage in migratory birds. These efforts align with the One Health approach, which recognizes the interconnectedness of human, animal, and environmental health. However, monitoring alone is insufficient. A comprehensive strategy demands international cooperation among public health experts, ecologists, veterinarians, and policymakers to mitigate the spread of zoonotic pathogens and AMR. Failure to implement coordinated measures could exacerbate global health threats, underscoring the urgency of integrating wildlife surveillance into One Health frameworks. These efforts must also account for confounding influences such as habitat, diet, seasonality, and anthropogenic exposure.

## Perspectives

Migration is a crucible of microbial transformation, where physiology, ecology, and environment converge to sculpt the avian gut microbiota. Yet, the forces driving this remodeling dietary flux, immunological strain, and environmental exposure remain largely unresolved. Importantly, current evidence remains fragmented across species and regions, and many proposed mechanisms reflect working hypotheses rather than universal rules. Bridging these gaps demands bold, integrative science. We propose multiple avenues for future research, categorized into five primary areas: (i) a conceptual synthesis of migration-driven microbiota adaptation and spillover, (ii) taxonomic expansion of avian species, (iii) functional validation, (iv) meta-omic resolution, and (v) translation into conservation and One Health practice. Pursued together, these goals will establish migratory birds as global sentinels of microbial adaptation and ecological spillover.

### Conceptual framework: gut microbiota adaptation and spillover pathways

Migratory birds show strong microbial plasticity, shaped by ecological and physiological pressures. The framework outlined here is intended as hypothesis generating, given limited longitudinal evidence and strong seasonal/confounding effects. Addressing these confounders requires longitudinal sampling across phases, paired environmental metadata (diet, habitat, season), and statistical approaches that separate migratory effects from seasonal variation. We hypothesize that migration induces sustained pressures on microbial communities, resulting in long-term microbial shifts that optimize host metabolism, immunity, and stress responses over the course of migration cycles. Stopover sites serve as microbial convergence points, where birds interact with local species and environments, facilitating microbial exchange, including zoonotic pathogens and AMR genes. Nonetheless, the strength and direction of these interactions vary widely among species and habitats, and longitudinal evidence directly linking migration to stable microbial restructuring is still sparse. This framework highlights migration as both an adaptive and evolutionary process that shapes gut microbial dynamics over time, across varied geographies, and over multiple migratory cycles.

### Knowledge extension of the microbiota for more avian taxa

Current understanding of gut microbial diversity in migratory birds is limited by the narrow range of species studied, leading to broad inferences that may not capture the full spectrum of avian gut microbiota diversity. Given the vast species richness and diverse life histories in birds, the microbiota is likely equally diverse. Unlike mammals, birds lack initial mechanical digestion, relying more on microbial processing [239], suggesting a uniquely central role for gut symbionts [41]. Future research should target underrepresented avian taxa and biogeographic regions through collaborative, multi-institutional projects. For instance, a study on neotropical birds and the Arctic Shorebird Demographics Network project illustrate successful collaborations that expanded the geographical and taxonomic scope of gut microbiota research [45]. Expanding species coverage will also help evaluate whether patterns proposed in conceptual models hold broadly or are restricted to certain ecological guilds.

### Functional validation for candidate microbiota from wild birds by a control experiment

While correlations between microbiota and host traits (e.g. fat storage, immunity) are well documented in poultry, experimental validation remains rare, especially in wild species. Functional studies using microbiota transfer, dietary manipulation, or antibiotic perturbation can test causality. Comparing wild and captive populations offers opportunities to explore how natural microbial communities respond to controlled environmental or dietary inputs. Short-term common garden or semi-wild enclosure experiments can help identify candidate taxa and metabolites that influence key migratory traits like thermogenesis, immune resilience, or energy metabolism. Such experiments are essential because observational field studies alone cannot disentangle migration effects from climate, resource availability, or temporal changes.

### Application of multi-omics technologies integrated with the biological context of migration

Recent advances in meta-omics, including metagenomics, metatranscriptomics, metaproteomics, and metabolomics, enable comprehensive functional profiling of the gut microbiota beyond taxonomy. Applying these approaches across the migratory cycle can help identify how physiological stressors such as heat, fasting, altitude, and circadian rhythms alter microbial function. Mapping microbial gene expression and metabolic outputs over time can reveal core functional strategies supporting migration, such as energy production, inflammation control, and tissue repair. Emphasizing functional over taxonomic profiling will offer deeper insights into microbial contributions to avian migration. Importantly, multi-omics datasets will also help detect whether proposed migration-linked microbial functions persist after controlling for confounding seasonal or environmental influences.

### Integration into conservation and disease mitigation

The global mobility of migratory birds makes them powerful indicators and transmitters of ecological and public health risks, including zoonotic pathogens and AMR genes. Embedding gut-microbiota surveillance into ringing stations, eBird platforms, and wildlife-health programs will enable early warning of spillover risk. A One Health framework integrating ecological, veterinary, and human health disciplines is essential for mitigating the impact of microbial spillover and preserving migratory species under growing pressures from climate change, habitat loss, and globalization. However, any conservation or disease-mitigation strategies must understand that birds represent only one component of broader transmission networks dominated by livestock systems, wastewater, and human movement, essential for balanced risk assessment.

## Conclusions

Migratory birds provide an invaluable natural model system for understanding how extreme physiological and ecological transitions reshape host–microbiota interactions. As birds move across continents, rapid changes in diet, environment, and immune activity remodel their gut microbiota in both taxonomic and functional dimensions. These shifts are not uniform across species, reflecting a mixture of conserved microbial functions and species-specific ecological strategies. Migration therefore acts both as a selective force shaping microbial adaptation and as a conduit for the global movement of pathogenic and antimicrobial-resistant microbes. These dynamics highlight the need for cross-disciplinary approaches integrating meta-omics, longitudinal tracking, and controlled experiments to clarify the mechanisms by which microbiota support avian health, immunity, and resilience. Despite their ecological importance, wild and migratory birds remain strikingly underrepresented in microbiome research. Closing this gap is essential for understanding how microbial symbionts contribute to host adaptation under rapidly changing environmental conditions and for anticipating how global change may alter host–microbe interactions with consequences for biodiversity, ecosystem stability, and zoonotic risk. A One Health framework, supported by advances in sequencing technologies and international collaboration, will be critical for translating these insights into effective conservation strategies and global-public-health preparedness.

## Supplementary Material

ycag042_Supplementary_Tables

## Data Availability

Data sharing is not applicable to this article as no datasets were generated or analyzed during the current study.
