# Supplementary material for: Migration-driven microbial adaptation and ecological spillover in birds
Source: ISME Commun. 2026 Mar 10;6(1):ycag042. doi: 10.1093/ismeco/ycag042 (PMC13037478; doi:10.1093/ismeco/ycag042)
Supplement: ycag042_Supplementary_Tables [file ycag042_supplementary_tables.docx]

| **Table S1: Gut Microbiota Compositional Shifts Across Migratory Phases** | | |
| --- | --- | --- |
| **Study Species** | **Key Study Focus** | **Source** |
| Blackpoll Warbler (*Setophaga striata*) | - ↓ Alpha diversity during migration - ↑ Proteobacteria - ↓ Firmicutes - ↑ Enterobacteriaceae family - Enrichment of pathways involved in vitamin, amino acid, and fatty acid biosynthesis, carbohydrate degradation, and homolactic fermentation of carbohydrates to the short-chain fatty acid lactate | [1] |
| Catharus spp. *(Catharus thrushes)* | - Microbiota composition varied more across seasons and years than between bird species - ↓ Alpha diversity during migration - The genera 67–14, Lysobacter, Babeliales, Nannocystaceae, and Ralstonia were significantly more abundant in birds during spring migration (vs breeding). - During fall migration, the genera 67–14, Ralstonia, Nannocystaceae, Babeliales, and Bacillus were significantly more abundant (vs breeding) | [2] |
| Kirtland's Warbler (*Setophaga kirtlandii*) | - Gut microbiota composition changes between the wintering grounds in The Bahamas and breeding territories in Michigan - Stable core taxa across phases - ↑ Alpha diversity in the wintering phase than in the Breeding - ↑ Bacteroidetes and Actinobacteria in the Bahamas - ↑Proteobacteria in Michigan | [3] |
| Hooded Crane (*Grus monacha*) | - ↓ Fungal alpha diversity at spring and autumn stopover sites compared to wintering grounds - ↑ Relative abundance of *Zygomycota* and *Rozellomycota* in wintering grounds compared to stopovers - ↑ Potentially pathogenic fungal taxa detected in wintering grounds relative to spring migration stopover sites | [4] |
| Relict Gull (*Ichthyaetus relictus*) | - ↑ Alpha diversity during wintering - ↓ Firmicutes, - ↑ Proteobacteria - Enhanced energy metabolism | [5] |
| Hooded crane (*Grus monacha*) | - ↓ Alpha diversity at stopovers (spring & autumn) vs wintering - A difference in the relative abundance of *Alphaproteobacteria* and *Lactobacillus* was observed between the wintering and stopover periods | [6] |
| Swainson’s thrush (*Catharus ustulatus*) & Grey catbird (*Dumetella carolinensis*) | - Substantial changes in gut microbiota composition were observed between spring and fall migratory birds - No specific gut microbial taxa were linked to body condition at the stopover site during either spring or fall migration | [7] |
| Black-necked Crane (Grus nigricollis) | - ↑ Alpha diversity in the wintering period than in the breeding period - The highest alpha and beta diversity of gut microbiota in summer - lower alpha diversity in spring than in autumn. - ↓ Alpha diversity during early migration - Strong seasonal restructuring of core taxa - ↑ Proteobacteria during migration; ↑ Firmicutes during winter - Predicted pathways enriched for amino-acid biosynthesis and energy metabolism in winter | [8] |
| White-tailed Eagle (Haliaeetus albicilla) | - ↓ Alpha diversity during autumn, ↑ Alpha diversity during spring migration - ↑ Firmicutes in winter; ↑ Bacteroidetes in breeding - Enrichment of lipid and carbohydrate metabolic pathways during migration | [9] |
| Himalayan Bluetail (Tarsiger rufilatus) | - Marked restructuring of microbiota with elevation - ↑ Alpha diversity during the spring season and ↓ in autumn at breeding sites (high elevation) - ↑ Alpha diversity during the autumn and ↓ spring seasons at non-breeding sites (low elevation) - ↑ Firmicutes, Bacteroidetes in Winter; ↑ Proteobacteria in Breeding correlated with a high-protein insect diet. - Carbohydrate metabolism pathways were enriched in Wintering, while in breeding, protein degradation and energy metabolism were higher. | [10] |

Notes: ↑ = increase; ↓ = decrease in microbial diversity, taxa, or functional pathways relative to comparison groups.

| **Table S2: Convergence with Local Microbiota at Stopovers** | | |
| --- | --- | --- |
| Garden Warbler (*Sylvia borin*) & Willow Warbler (*Phylloscopus trochilus*) | - Gut microbiota composition at breeding and wintering grounds mirrored resident species - Genera such as *Staphylococcus* and *Serratia* showed marked shifts between breeding and wintering phases | [11] |
| Ruddy Turnstone (*Arenaria interpres*) | - Alpha diversity increased over time post-arrival at the staging site - The abundance of *Vibrio* and *Flavobacterium* was associated with variations in body weight - Functional gene profiles differed among individuals in different weight categories - Expression of metabolic genes related to polyunsaturated fatty acid synthesis was associated with weight gain | [12] |
| Swainson’s Thrush (*Catharus ustulatus*), Wood Thrush (*Hylocichla mustelina*), Gray Catbird (*Dumetella carolinensis*) | - Sampled individuals shortly after arrival at the Gulf Coast stopover - Gut microbiota initially varied post-arrival but rapidly converged to resemble resident birds within days, indicating environmental filtering | [13] |
| Black-necked Crane (Grus nigricollis) | - ↑Alpha diversity in winter - Winter microbiota resembled long-resident populations | [8] |
| Migratory Shorebirds | - Stopover habitat selection strongly predicts gut microbiome composition - Rapid acquisition of local microbial signatures - Age-related ↑ alpha diversity during stopover | [14] |
| Relict Gull (*Larus relictus*) | - ↑ Alpha Diversity at wintering grounds than at stopovers - Microbiota exhibit convergence driven by seasonal diet changes at the stopover site. - ↓ Firmicutes, ↑ Proteobacteria in winter | [15] |

Notes: ↑ = increase; ↓ = decrease in microbial diversity, taxa, or functional pathways relative to comparison groups.

| **Table S3: Comparative Insights: Migrant vs. Resident Birds** | | |
| --- | --- | --- |
| Curlew sandpiper (*Calidris ferruginea*) and Red-necked stint (*Calidris ruficollis*) | - Migrants exhibited higher levels of Actinobacteria compared to the resident species - *Corynebacterium* was especially abundant in migrants across multiple species and locations - Apart from differences in *Corynebacterium*, migration had only a modest influence on overall gut microbiota composition - Variation in gut microbiota between migrants and residents reduced as the time between migrant arrival and sampling increased | [16, 17] |
| Barn swallow (*Hirundo rustica*) | - Significant differences in beta diversity were observed between migratory and resident individuals - Migrants had notably higher levels of the genera *Catellicoccus*, *Clostridium* *sensu* *stricto* 1, *Citrobacter*, *Corynebacterium*, *Fusobacterium*, *Lactobacillus*, *Leuconostoc*, *Mycoplasma*, *Romboutsia*, *Staphylococcus*, and *Turicibacter* compared to residents - ↑ Firmicutes in migrants compared to resident individuals | [18] |
| Canada geese (*Branta canadensis*) | - ↓ alpha diversity in migrants - The genera *Epulopiscium*, *Cellulosilyticum*, *Terrisporobacter*, and *Turicibacter* were among the most differentially abundant in migrants - ↑ Firmicutes in migratory individuals - Notably, resident populations were sampled from urban environments, potentially introducing confounding effects in comparisons between migratory and resident microbiomes | [19] |
| Red-headed bunting (*Emberiza bruniceps*) | - Gut microbiota composition differed in association with the migratory phenotype - The data suggested a relationship between microbiome shifts and blood metabolites linked to migratory behaviour, although a direct causal connection was not demonstrated | [20] |
| High-Altitude Passerines (Qinghai–Tibet plateau) | - Migrants exhibit convergent gut microbiome features (functional and taxonomic) distinct from non-migrants | [10] |

Notes: ↑ = increase; ↓ = decrease in microbial diversity, taxa, or functional pathways relative to comparison groups.

**Reference**

1. Trevelline BK, Sprockett D, DeLuca WV *et al.* Convergent remodelling of the gut microbiome is associated with host energetic condition over long‐distance migration. *Functional ecology*. 2023;**37**:2840-54

2. Skeen HR, Willard DE, Jones AW *et al.* Intestinal microbiota of nearctic‐neotropical migratory birds vary more over seasons and years than between host species. *Mol Ecol*. 2023;**32**:3290-307

3. Skeen HR, Cooper NW, Hackett SJ *et al.* Repeated sampling of individuals reveals impact of tropical and temperate habitats on microbiota of a migratory bird. *Mol Ecol*. 2021;**30**:5900-16

4. Mahtab N, Zhou L, Zhang F *et al.* Seasonal variations in the gut fungal communities of hooded crane (grus monacha) at wintering and stopover sites in china. *Animals*. 2021;**11**:941

5. Yao H, Zhang Z, Wu N *et al.* Comparative analysis of intestinal flora at different overwintering periods in wild relict gulls (larus relictus): First evidence from northern china. *Frontiers in Microbiomes*. 2023;**2**:1218281

6. Zhang F, Xiang X, Dong Y *et al.* Significant differences in the gut bacterial communities of hooded crane (grus monacha) in different seasons at a stopover site on the flyway. *Animals*. 2020;**10**:701

7. Lewis WB, Moore FR, Wang S. Characterization of the gut microbiota of migratory passerines during stopover along the northern coast of the gulf of mexico. *J Avian Biol*. 2016;**47**:659-68

8. Zhang Y, Ma R, Suolangduoerji *et al.* Annual cycle variations in the gut microbiota of migratory black-necked cranes. *Frontiers in microbiology*. 2025;**16**:1533282 <https://doi.org/10.3389/fmicb.2025.1533282>

9. Ouyang X, Guan Y, Pei J *et al.* Seasonal variation in gut microbiota of migratory wild raptors: A case study in white-tailed eagles. *Animal microbiome*. 2025;**7**:37 <https://doi.org/10.1186/s42523-025-00406-y>

10. Zhang S, Zhou C, Dong Z *et al.* The diet-intestinal microbiota dynamics and adaptation in an elevational migration bird, the himalayan bluetail (tarsiger rufilatus). *Ecology and evolution*. 2024;**14**:e11617 <https://doi.org/10.1002/ece3.11617>

11. Schmiedová L, Kreisinger J, Kubovčiak J *et al.* Gut microbiota variation between climatic zones and due to migration strategy in passerine birds. *Frontiers in Microbiology*. 2023;**14**:1080017

12. Grond K, Louyakis AS, Hird SM. Functional and compositional changes in the fecal microbiome of a shorebird during migratory stopover. *MSystems*. 2023;**8**:e01128-22

13. Lewis WB, Moore FR, Wang S. Changes in gut microbiota of migratory passerines during stopover after crossing an ecological barrier. *The Auk: Ornithological Advances*. 2017;**134**:137-45

14. Włodarczyk R, Drzewińska-Chańko J, Kamiński M *et al.* Stopover habitat selection drives variation in the gut microbiome composition and pathogen acquisition by migrating shorebirds. *FEMS microbiology ecology*. 2024;**100** <https://doi.org/10.1093/femsec/fiae040>

15. Wu H, Yao H, Sun M *et al.* Inter-year consistencies and discrepancies on intestinal microbiota for overwintering relict gulls: Correlations with food composition and implications for environmental adaptation. *Frontiers in microbiology*. 2024;**15**:1490413 <https://doi.org/10.3389/fmicb.2024.1490413>

16. Risely A, Waite DW, Ujvari B *et al.* Active migration is associated with specific and consistent changes to gut microbiota in calidris shorebirds. *Journal of Animal Ecology*. 2018;**87**:428-37

17. Risely A, Waite D, Ujvari B *et al.* Gut microbiota of a long‐distance migrant demonstrates resistance against environmental microbe incursions. *Mol Ecol*. 2017;**26**:5842-54

18. Turjeman S, Corl A, Wolfenden A *et al.* Migration, pathogens and the avian microbiome: A comparative study in sympatric migrants and residents. *Mol Ecol*. 2020;**29**:4706-20

19. Obrochta S, Savo Sardaro ML, Amato KR *et al.* Relationships between migration and microbiome composition and diversity in urban canada geese. *Frontiers in Ecology and Evolution*. 2022;**10**:742369

20. Gupta NJ, Das S, Das M *et al.* Seasonal plasticity and energy efficiency in migratory buntings is an assemblage of alterations in metabolic and gut microbe adaptations. *bioRxiv*. 2023:2023.02. 17.529037
